# Comparative virulence of Caribbean, Brazilian and European isolates of *Toxoplasma gondii*

**DOI:** 10.1186/s13071-019-3372-4

**Published:** 2019-03-14

**Authors:** Clare M. Hamilton, Lauren Black, Solange Oliveira, Alison Burrells, Paul M. Bartley, Renata Pimentel B. Melo, Francesca Chianini, Javier Palarea-Albaladejo, Elisabeth A. Innes, Patrick J. Kelly, Frank Katzer

**Affiliations:** 1 Moredun Research Institute, Pentlands Science Park, Midlothian, EH26 0PZ UK; 2 Department of Preventive Veterinary Medicine and Animal Health, School of Veterinary Medicine, University of São Paulo, São Paulo, SP CEP 05508-000 Brazil; 3 Department of Veterinary Medicine, Laboratory of Infectious-Contagious Diseases of Domestic Animals, Universidade Federal Rural de Pernambuco, Rua Dom Manoel de Medeiros, Recife, PE 52171-900 Brazil; 4 Biomathematics & Statistics Scotland, JCMB, The King’s Buildings, Edinburgh, EH9 3FD Scotland, UK; 5 Ross University School of Veterinary Medicine, Island Main Road, West Farm, Basseterre, Saint Kitts and Nevis

**Keywords:** *Toxoplasma gondii*, Virulence, Genotyping, Isolates, Caribbean, Brazil, Europe

## Abstract

**Background:**

*Toxoplasma gondii* is a zoonotic parasite of global importance. The outcome of infection in humans can depend on a number of factors including the infecting stage of the parasite, inoculating dose and virulence of the infecting strain. Molecular epidemiological studies have demonstrated an abundance of atypical strains of *T. gondii* in South America, many of which have been associated with more severe sequelae of infection. The aim of this study was to compare the virulence of *T. gondii* strains isolated in the Caribbean to a virulent Brazilian strain and an avirulent European strain.

**Methods:**

One hundred and twenty Swiss CD-1 mice were split into 8 groups of 15 mice and each group was inoculated with 200 tachyzoites of one of 8 isolates, comprising ToxoDB genotypes #1, #141, #265, #13, #3 and #6. Five mice per group were euthanized at day 8 post-inoculation (p.i.) and parasite burden was determined in heart, lungs and eyes using quantitative PCR. Lungs and brain were also examined by histopathology and immunohistochemistry. The remaining 10 mice per group were part of a survival experiment to assess virulence. DNA was extracted from tachyzoites of each of the 8 *T. gondii* isolates and genotyped at four ROP gene loci, including ROP5, ROP16, ROP17 and ROP18 to look for association with markers of virulence.

**Results:**

Infection with ToxoDB genotype #13 from the Caribbean resulted in 100% of mice being euthanized which was comparative to infection with the virulent Brazilian strain (ToxoDB genotype #6). Significantly higher parasite burdens were recorded in the lungs and eyes of mice infected with ToxoDB genotypes #13 and #6. Genotyping of ROP loci revealed that the virulent Caribbean isolates had a different ROP18/ROP5 allelic profile (3/1) to the virulent Brazilian isolate (1/3); however, the avirulent Caribbean isolate (ToxoDB genotype #1) had the same ROP18/ROP5 profile as the avirulent European isolate (ToxoDB #3) (both 2/2). Caribbean isolates of intermediate virulence (ToxoDB #141 and #265) all had the same ROP18/ROP5 allelic profile (2/2).

**Conclusions:**

Isolates from the Caribbean with ToxoDB genotype #13 were acutely virulent for mice and comparable to a known virulent Brazilian isolate. The ROP protein allelic profile of the virulent Caribbean and Brazilian isolates differed indicating that perhaps other factors are involved in predicting virulence. Understanding virulence is important for predicting disease outcome in humans and may also aid vaccine design as well as drug discovery.

## Background

*Toxoplasma gondii* is a ubiquitous intracellular parasite capable of infecting all warm-blooded animals, including humans [1]. Felids are the only definitive hosts shedding large numbers of oocysts into the environment in their faeces. Transmission routes of the parasite to humans include ingestion of infectious oocysts directly from the environment or in contaminated food or water, ingestion of tissue cysts in undercooked/raw infected meat, and vertical transmission from mother to foetus during a primary infection. Infection in immune competent people is usually mild or asymptomatic; however, immune compromised people and congenitally infected infants can suffer severe and life-threatening clinical signs, such as encephalitis, blindness or even death [2].

The population structure of *T. gondii* was originally thought to be clonal, consisting of three dominant lineages (designated Types I, II and III) [3]; however, it has become apparent that the parasite has much greater genetic variability and some genotypes are distributed in distinct geographical patterns [4]. In Europe and North America, clonal Types II and III dominate (as well as Type 12 in North America), whilst in South and Central America there is an abundance of atypical (non-clonal) strains with no clear genotypes dominating [4]. Virulence of *T. gondii* has been assessed in mice and varies depending on the infecting strain, with Type I genotypes causing 100% mortality irrespective of the inoculating dose and Types II and III causing intermediate or no mortality, depending on dose [5]. A recent study correlating virulence markers with published virulence data for over 200 isolates from all over the world noted that isolates from North America, Europe, North Africa and Asia were non-lethal to mice at low infectious doses whereas a large proportion of isolates from South America were lethal to mice [6].

Polymorphic rhoptry proteins (ROP) are secreted by the parasite upon invasion and have been shown to be involved in host-parasite interactions and immune evasion [7]. Recently, ROP5 and ROP18 alleles were identified as key determinants of virulence in mice [6]. Studies on the population genetics and epidemiology of *T. gondii* have suggested a link between the geographical distribution of isolates and more severe clinical signs in immune competent humans. In South America (specifically, French Guiana and Suriname), there have been reports of severe systemic toxoplasmosis resulting in death of immune competent people infected with an atypical strain of the parasite [8]. Similarly, the prevalence, severity and risk of ocular toxoplasmosis is much greater in Brazil than in North America and Europe [9].

The virulence of *T. gondii* strains in outbred mice appears to correlate with disease manifestations in humans making the mouse a useful tool for assessing virulence and predicting possible outcomes of human infection [10, 11]. The aim of this study was to determine the virulence of previously identified atypical strains of *T. gondii* isolated in the Caribbean [12] and compare them with a known virulent Brazilian strain [13] and an avirulent European strain [14].

## Methods

### *In vitro* culture of tachyzoites

*Toxoplasma gondii* tachyzoites of the strains listed in Table 1 were grown in pre-cultured Vero cells (ATCC® CCL-81™) in IMDM (supplemented with 2% FCS, 200 IU/ml penicillin and 200 μg/ml streptomycin) at a ratio of one Vero cell to two *T. gondii* tachyzoites. On the day of inoculation, tachyzoites were enumerated using a haemocytometer and one extra vial per group (Table 1) was prepared and stored at -20 °C for DNA extraction and confirmation of genotype by PCR-RFLP.

**Table 1:** Isolates and groupings for experimental infection with 200 *T. gondii* tachyzoites of different genotypes

Group no.	Inoculating isolate	Origin of isolate	ToxoDB RFLP genotype #	No. of mice	Day p.i. of intended euthanasia	Reference
1	TgCkStk12	St. Kitts	1 (type II)	5	8	[12]
				10	28	
2	TgCkStK13		141 (atypical)	5	8	
				10	28	
3	TgCkStK10		141 (atypical)	5	8	
				10	28	
4	TgCkStK2		265 (atypical)	5	8	
				10	28	
5	TgCkStK9		13 (atypical)	5	8	
				10	28	
6	TgCkStK11		13 (atypical)	5	8	
				10	28	
7	Moredun M4	UK	3 (type II variant)	5	8	[15]
				10	28	
8	TgCatBr71	Brazil	6 (atypical)	5	8	[13]
				10	28	

### Mouse virulence study

One hundred and twenty female outbred Swiss CD-1 mice were housed in groups of five with access to food and water *ad libitum*. The mice were split into 8 groups of 15 mice. Within each group, 5 mice were euthanised on day 8 post-inoculation (p.i.) to allow for comparison of parasite burden and distribution at one time-point, and 10 mice were part of a survival experiment to assess virulence of the isolates. All mice were inoculated, intraperitoneally, with 200 tachyzoites (Table 1). Although only a single dose was chosen; this was based on previous experiments demonstrating that virulent isolates resulted in 100% mortality irrespective of dose [15–18] and that avirulent and intermediately virulent isolates may also be identified at a lower dose [19, 20]. Mice were monitored and scored twice daily (at least) for signs of clinical toxoplasmosis. Any mice that reached a maximum permissible score for 2 consecutive days and were deemed unlikely to recover were humanely euthanised in accordance with the Animals (Scientific Procedures) Act 1986. Remaining mice were euthanised when they reached the end of the experiment (day 8 p.i. or day 28 p.i.). Immediately following euthanasia, mice were bled by cardiac puncture and sera isolated as previously described [12]. One cerebral hemisphere, one lung and both eyes from each mouse were transferred to clean 2 ml tubes and stored at -80 °C for DNA extraction, while one cerebral hemisphere and one lung were stored in 10% buffered formalin for histopathology examination (described below). All sera from mice in the survival experiment (10 per group) were tested for antibodies to *T. gondii* using an indirect ELISA (ID Screen® Toxoplasmosis Indirect Multi-species, IDvet, Montpellier, France) according to the manufacturer’s instructions. Sera from the 5 mice per group which were euthanised at day 8 p.i. were not tested as the ELISA detects IgG and these samples would have been negative (too early in infection).

### DNA extraction and quantitative PCR

One cerebral hemisphere from each mouse was homogenised in 1 ml PBS using a syringe and 18G needle followed by a 21G needle. One lung from each mouse was homogenised in 1 ml Nuclei Lysis Solution (Promega Corporation, Southampton, UK) in Precellys tubes containing CK28 ceramic beads. Both eyes from each mouse were homogenised, together, in 1 ml Nuclei Lysis Solution using an 18G needle. DNA was extracted from 400 μl of each homogenised tissue using the Wizard® genomic DNA purification protocol (Promega Corporation, Southampton, UK) [21]. The final pellet of DNA was resuspended in 200 μl nuclease-free water and stored at -20 °C until required for PCR.

Quantitative PCR, targeting the 529 bp repeat element, was carried out in triplicate according to a previously described method [22] with slight modifications. The 20 μl reaction mixture consisted of 10 μl 2× Lightcycler® 480 Probes Master mix (Roche), 0.7 μM of each primer (Tox-9Fand Tox-11R), 0.1 μM of Tox-TP1, 0.2 μM of CIAC-probe, 0.02 fg of CIAC and 250 ng of template DNA in 8 μl. Sequences for primers and probes, as well as PCR conditions have been described previously [12, 22].

### Genetic characterisation of *T. gondii* by PCR-RFLP

DNA was extracted from aliquots of mouse inocula (tachyzoites representing each isolate) using proteinase K (Promega). Briefly, 100 μg/ml proteinase K was added to each sample prior to incubation at 50 °C for 90 min, followed by 95 °C for 15 min. Samples were centrifuged at 13,000× *g* for 5 min at 4 °C and the supernatant (containing the DNA) was transferred to a clean 2 ml tube and stored at -20 °C until required. The ToxoDB genotype of each isolate was confirmed using a multiplex nested PCR-RFLP targeting 10 genetic markers, including SAG1, SAG2 (5′- and 3′-SAG2 and Alt.SAG2), SAG3, BTUB, GRA6, c22-8, c29-2, L358, PK1 and Apico as previously described [23, 24]. Genotypes were determined using RFLP banding profiles of reference strains RH (Type I), M4 (Type II variant; used for all markers except Apico where Type II strain Me49 was used instead) and NED (Type III) [25].

Genotyping of loci known to be associated with virulence (ROP5, ROP16 and ROP18) was carried out using PCR-RFLP methods as previously described [6, 24]. For ROP18, two sets of primers were used: one to amplify a repetitive sequence (DEL) in the promoter region of archetypal Type I and II alleles; and one to amplify the upstream promoter insertion sequence (UPS) of the archetypal Type III allele [6]. As the UPS fragment is only amplified for Type III alleles no restriction digest was required. A double digest was performed on the DEL fragment and banding patterns were compared to reference strains RH and M4 [6].

### Histopathology and immunohistochemistry

Formalin-fixed samples of brain and lung were processed routinely and embedded in paraffin wax. A formalin fixed paraffin-wax embedded liver from a mouse known to be infected with *T. gondii* was used as a positive control. Sections were cut at 5 µm and stained with haematoxylin and eosin (HE) for histological examination. For immunohistochemistry (IHC), serial sections were cut at 5 µm and placed on treated glass slides (Superfrost Plus, Menzel-Glaser, Braunschweig, Germany). For IHC, tissues were deparaffinised and hydrated before endogenous peroxidase was blocked with 3% hydrogen peroxide and methanol solution for 20 min at room temperature. For heat-induced epitope retrieval (HIER), slides were placed into citrate buffer (0.01M citric acid, pH6) and then autoclaved for ten minutes at 121 °C. Normal goat serum (25%) was applied to all slides for at least 30 min at room temperature. *Toxoplasma gondii* rabbit polyclonal antibody (Thermo-Fisher Scientific, Illinois, USA), diluted 1:600, was applied to all samples overnight at 4 °C. For the negative control, normal rabbit serum (HKV1) was used at 1:500. After 18–24 hours, goat anti-rabbit HRP-labelled polymer (DAKO EnVision+, Carpinteria, California, USA) was applied to the slides for 30 min at room temperature. AEC (3-amino-9-ethylcarbazole) chromogen solution (Vector Laboratories, Peterborough, UK) was made as instructed in the manufacturers guidelines and applied to slides for 30 min at room temperature. All tissues were counterstained with hematoxylin, then mounted with Marienfeld microscope cover slips (Lauda-Königshofen, Germany) using Shandon Consul-Mount™ Histology Formulation (Xylene based). Sections of organ samples collected from the eight groups of mice were assessed microscopically and the severity of pathology was based on the number and size of lesions observed. The presence of *T. gondii* was assessed by immunohistochemistry.

### Statistical analysis

The virulence of the isolates was assessed by estimating survival curves using the Kaplan-Meier method based on 10 mice per group. The overall difference between survival curves was tested for statistical significance using the asymptotic log-rank test for multiple groups [26] based on a chi-square probability distribution with 7 degrees of freedom. This was followed by *post-hoc* pairwise comparisons between curves using 2-group log-rank tests, with the resulting *P*-values being corrected for multiple comparisons using the Benjamini-Hochberg’s procedure [27] to control for false discovery rate (FDR).

Parasite burden in lung, brain and eyes was measured using a quantitative PCR and the data were analysed using a linear mixed model (LMM) fitted by restricted maximum likelihood (REML) to log-transformed data, with “Group” and “Organ”, and the interaction between them, as fixed effects and “Animal” as a random effect. *Post-hoc* pairwise comparisons between organs and groups were conducted from the LMM estimates, with the corresponding *P*-values being adjusted for FDR.

Statistical test significance was assessed at the usual 5% significance level. The statistical analyses were conducted on the R system for statistical computing v3.4 [28].

## Results

### Mouse survival experiment

**Fig. 1:**
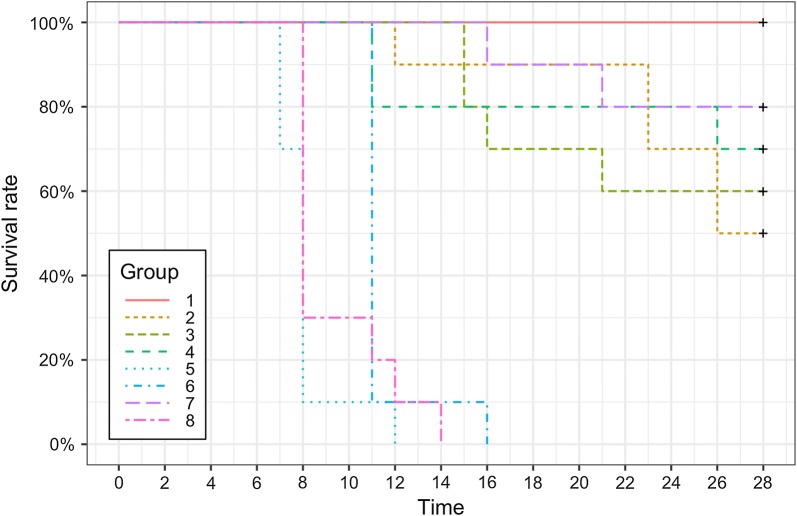
Kaplan-Meier surival curves for each group of mice infected with one of 8 isolates of *T. gondii* (*n* = 10 per group). All mice (100%) in Group 1 surived until the end of the experiment (day 28 p.i.), whereas no mice (0%) survived in Groups 5, 6 and 8. Mice in Groups 2, 3, 4 and 7 had survival rates of 50%, 60%, 70% and 80%, respectively

During the 28-day survival experiment, only 45% (36/80) of mice survived until the end of the experiment (Fig. 1). Forty-four mice (55%) had to be euthanised due to signs of clinical toxoplasmosis. Of the 44 mice euthanised, 20 had been infected with ToxoDB genotype #13 (St. Kitts isolates TgCkStK9 and TgCkStK11; Groups 5 and 6, respectively), 10 with ToxoDB genotype #6 (Brazilian BrI isolate TgCatBr71; Group 8), 9 with ToxoDB genotype #141 (5 with St. Kitts isolate TgCkStK13 and 4 mice with St. Kitts isolate TgCkStK10; Groups 2 and 3, respectively), 3 with ToxoDB genotype #265 (St. Kitts isolate TgCkStK2; Group 4) and 2 with ToxoDB genotype #1 (M4; Group 7). Kaplan-Meier survival curves were plotted to present the survival rate of mice for each group (Fig. 1). A log-rank test demonstrated an overall statistically significant difference in survival between all groups of mice (*χ*^2^ = 54.07, *df* = 7, *P* < 0.001). Pairwise comparisons revealed that mice infected with ToxoDB genotype #13 (Groups 5 and 6) and ToxoDB genotype #6 (Group 8) had a significantly lower survival rate in comparison to all other groups (*P* < 0.001). Mice infected with ToxoDB genotype #1 (Group 1) had a statistically significantly higher survival rate compared to all other groups of mice (*P* < 0.05) except Group 7 (European M4 isolate; ToxoDB genotype #3) and Group 4 (ToxoDB genotype #265) (*P* = 0.019 and *P* = 0.094, respectively).

### Distribution and burden of parasites

**Fig. 2:**
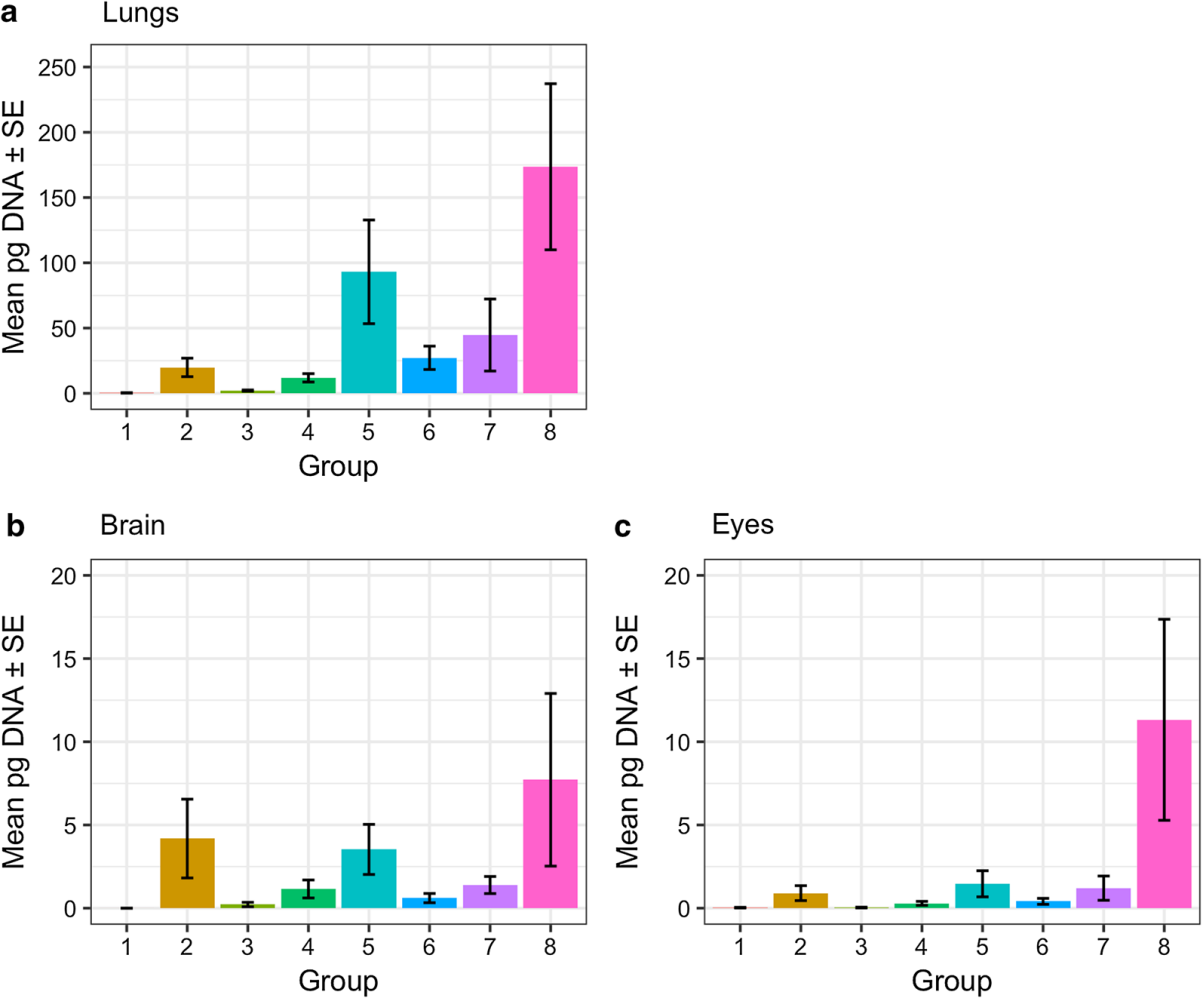
Parasite burden in mouse tissues. Mean quantity (± SE) of *T. gondii* DNA, as detected by the 529 bp repeat element qPCR, in the lungs (**a**), brain (**b**) and eyes (**c**) of mice euthanized at day 8 p.i. (*n* = 5 per group)

Parasite distribution and burden were investigated in five mice from each group euthanised at day 8 post-infection. Using the 529-bp qPCR, *T. gondii* DNA was detected in the lungs of 100% (40/40) of mice, in the brain tissue of 77.5% (31/40) of mice and in the eyes of 80% (32/40) of mice. At day 8 p.i., there was a statistically significantly higher mean parasite burden in the lungs compared to brain and eyes (*F*_(2,63)_ = 126.71, *P* < 0.001; Fig. 2). The highest burden in the lungs (Fig. 2a) was evident in mice in Group 8 inoculated with the Brazilian BrI isolate (average *T. gondii* DNA concentration of 173.6 pg) followed by mice in Group 5 inoculated with the St. Kitts ToxoDB genotype #13 (average *T. gondii* DNA concentration of 93.1 pg). Mice in Group 1, inoculated with the Type II St. Kitts isolate TgCkStK12 (ToxoDB genotype #1), demonstrated the lowest lung burden with an average *T. gondii* DNA concentration of only 0.4 pg. Similar to the lung, the highest parasite burden in the brain (Fig. 2b) was observed in mice in Group 8 (average *T. gondii* DNA concentration of 7.7 pg), followed by Group 2 (average *T. gondii* DNA concentration of 4.2 pg) and then Group 5 (average *T. gondii* DNA concentration of 3.5 pg). The parasite burden in the eyes (Fig. 2c) was low compared to lung and brain. Again, the highest burden was observed in mice in Group 8 (11.3 pg), followed by Group 5 (1.5 pg) and then Group 7 (1.2 pg). When comparing parasite burden as a whole, the overall levels were generally higher in Groups 5 and 8, representing mice infected with ToxoDB genotype #13 (St. Kitts isolate TgCkStK9) and ToxoDB genotype #6 (Brazilian isolate TgCatBr71), respectively. For Group 8, the overall parasite burden was statistically significantly higher that all other groups (*P* < 0.001), except for Group 5 (*t*_(32)_ = -1.48, *P* = 0.173) and marginally Groups 2 and 7 (*t*_(32)_ = -2.28, *P* = 0.054; *t*_(32)_ = 2.22, *P* = 0.055). The pattern of differences in mean parasite burden between groups did not change statistically significantly across organs (*F*_(14,63)_ = 1.55, *P* = 0.120).

### Genetic characterisation and markers for virulence

Analysis of each of the 8 isolates by PCR-RFLP targeting 10 genetic markers (SAG1, SAG2 (5’, 3’ and Alt.), SAG3, BTUB, GRA6, c29-2, c22-8, L358, PK1 and Apico) revealed their expected genotypes [12] confirming that they had not changed during *in vitro* culture of tachyzoites for inoculation (data not shown). Results of PCR-RFLP analysis of polymorphic loci ROP5, ROP16, ROP17 and ROP18 are given in Table 2. For ROP5 and ROP18, three different alleles were identified, with allele 3 predominating for both loci, particularly ROP18. Loci ROP16 and ROP17 were less polymorphic with just two alleles being identified, although allele 1 predominated for both. Both the virulent isolates from St. Kitts (ToxoDB genotype #13; Groups 5 and 6) displayed a different profile to the virulent isolate from Brazil (ToxoDB genotype #6; Group 8). Of the 3 isolates of intermediate virulence, two isolates (St. Kitts isolates TgCkStK13 and TgCkStK10) displayed the same allelic profile (alleles 3, 3, 1, 1 for ROP5, ROP18, ROP16 and ROP17, respectively) and the other (St. Kitts isolate TgCkStK2) differed at the ROP17 locus (allele 2 rather than allele 1).

**Table 2:** Genotyping of isolates with polymorphic loci ROP5, ROP18, ROP16 and ROP17

Group	Isolate	ToxoDB genotype #	ROP5	ROP18	ROP16	ROP17	Virulence	% mortality
1	TgCkStk12	1	2	2	2	2	Avirulent	0
2	TgCkStK13	141	3	3	1	1	Intermediate	50
3	TgCkStK10	141	3	3	1	1	Intermediate	40
4	TgCkStK2	265	3	3	1	2	Intermediate	30
5	TgCkStK9	13	1	3	1	1	Virulent	100
6	TgCkStK11	13	1	3	1	1	Virulent	100
7	Moredun M4	3	2	2	2	2	Avirulent	20
8	TgCatBr71	6	3	1	1	1	Virulent	100

### Serology

All mice in the survival experiment which were euthanised later than day 16 p.i. (44/80) had detectable anti-*T. gondii* IgG antibodies in their sera (data not shown). Mice in the survival experiment which were euthanised earlier than this time point were negative by ELISA (data not shown).

### Histopathology

Lung and brain tissue from five mice from each group euthanised at day 8 p.i. were examined. Inflammatory infiltrate and necrotic foci were present in the lungs of all mice examined but these were more severe in the mice in Group 5 (St. Kitts isolate TgCkStK9, ToxoDB genotype #13). Mice from Groups 5, 6 and 8 all showed large numbers of parasite in the lungs. Although the lungs from mice in Group 8 displayed the highest burden of parasites, the pathology observed was not as severe as in Group 5. Very low numbers of parasites and mild/no pathology were seen in the brains of infected mice, which likely reflects the early time point of sample collection (day 8 p.i.).

## Discussion

In the present study, two isolates from St. Kitts, which were both ToxoDB genotype #13, proved to be highly virulent for mice and showed a level of pathogenicity similar to that of the known virulent Brazilian isolate BrI (ToxoDB genotype #6). All mice infected with these isolates (Groups 5, 6 and 8) showed severe signs of clinical toxoplasmosis, with maximum permissible scores for 2 consecutive days, and 100% were euthanised by days 12, 14 and 16, respectively for Groups 5, 8 and 6. Mice in Groups 5 and 8 also had higher parasite burdens in their lungs compared to other mice and mice in Group 5 had higher inflammatory infiltrate and necrotic foci in comparison to mice from the other groups. We have previously reported the potential virulence of ToxoDB genotype #13 in a bioassay where mice were inoculated with *T. gondii*-infected chicken tissues and 8 out 9 mice which received this genotype had to be euthanised due to clinical toxoplasmosis [12]. It is noteworthy that the findings in the present study are in contrast to previous studies involving ToxoDB genotype #13 which have all described the isolate as avirulent for mice [20, 29–35]. In six of the eight studies, virulence of the isolates was determined following a bioassay where tissues from dogs [30], chickens [31, 33], cats [32], goats [20, 35] and a howler monkey [34] were homogenised and inoculated into mice with no prior determination of parasite burden in the tissues. Therefore, it is possible that the inoculating dose may have been lower than that used in the present study and resulted in a less severe infection. In the present study, two different isolates of genotype #13 were used and both resulted in 100% mortality confirming the virulence of this isolate in this study. *Toxoplasma gondii* isolates of the same ToxoDB genotype but different levels of virulence have been described previously. Rego et al. [20] described two isolates (TgPgBrPI 8 and TgPgBrPI 1), both designated ToxoDB genotype #163, which resulted in 100% mortality and 0% mortality, respectively, in mice infected with 1, 10, 100 or 1000 tachyzoites. Also, isolates with the same ToxoDB genotype (#7) have been described as 100% virulent in one study [20] and of intermediate virulence in another [36] despite both studies using a titrated dose of tachyzoites.

ToxoDB genotype #13 has been isolated from a patient from the Caribbean with toxoplasmic lymphadenopathy and a history of toxoplasmic encephalitis [37] and phylogenetic analysis revealed that the strain clustered more closely with Type I strains than other strains [38]. This isolate (PSP-2003-ERO) had originally been genotyped using microsatellite markers and was defined as “Caribbean 1” genotype [39]. Caribbean 1 genotype (genotyped using microsatellite markers) has also been isolated at post mortem from an AIDS patient with toxoplasmic encephalitis and a transplant patient who died from pulmonary toxoplasmosis [39] and although these isolates remain to be genotyped using the panel of PCR-RFLP markers, it is highly likely that they are also ToxoDB genotype #13 [12]. This further highlights the potential virulence of this genotype. Indeed, it has been demonstrated that *T. gondii* isolates containing many Type I alleles are more pathogenic and likely to cause more severe disease [11, 40]. ToxoDB genotype #13 has Type I alleles at 5 out of 10 of the PCR-RFLP markers used to define it. It is also amongst the top ten most frequently isolated genotypes in South America [4].

Of note in the present study, was the burden of *T. gondii* in the eyes of mice infected with the Brazilian BrI isolate (Group 8). These mice had a statistically significantly higher burden in comparison to most of the other groups. BrI (ToxoDB genotype #6) is the second most frequently occurring isolate in Central and South America [4], and severe ocular toxoplasmosis has been shown to be diagnosed more often in this part of the world. For example, ocular involvement in *T. gondii-*infected patients in Europe and the USA has been estimated at 2% in comparison to almost 18% in Brazil [11, 41, 42]. Furthermore, congenitally infected children in Brazil are five times more likely to develop ocular lesions than European children, and the lesions are more severe and likely to lead to visual impairment [43].

ToxoDB genotype #141 proved to be of intermediate virulence in the present study, resulting in 50% and 40% mortality of mice in Groups 2 and 3, respectively. This genotype was previously isolated from a fox in the USA and was shown to be acutely virulent for mice even at low doses [18]. In contrast, in another study, this genotype was isolated from cats in St. Kitts (Caribbean) and was shown to be avirulent for mice [32]. In the latter study, the determination of virulence was based on a bioassay involving the inoculation of mice with digested tissues from seropositive cats and not a controlled study with a defined dose of parasites (as in the former study). This could explain the discrepancy between the results and highlights the potential pitfalls in assigning virulence based on bioassays in which the dose is not controlled, as outlined above for ToxoDB genotype #13. Differences in virulence may also be a reflection of the methods used to assign genotypes. Whilst PCR-RFLP is a widely used method, it is based on restriction enzymes detecting single nucleotide polymorphisms at a limited number of loci so it is possible that isolates which appear to have the same ToxoDB genotype may display undetected polymorphisms at other loci. Perhaps multi-locus sequence typing of virulence markers would offer a more robust approach, although this is more costly and time-consuming [11].

Rhoptry proteins (ROP) have been shown to play an important role in host-parasite interactions. They are secreted by the rhoptry bulb of *T. gondii* upon invasion of the host cell and act at the parasitophorous vacuole to inhibit the localisation of immunity-regulated GTPases (IRGs) and guanylate-binding proteins (GBPs), both of which have been shown to be host resistance factors to pathogens [44, 45]. Genetic crosses between the archetypal lineages of *T. gondii* and the subsequent analysis of their progeny resulted in the identification of ROP18 and ROP5 as key determinants of virulence in mice [46, 47]. Two other proteins, ROP16 and ROP17, have also been shown to play a role in the modulation of host responses to *T. gondii* invasion and the avoidance of parasite clearance [48, 49]. In a recent study, 240 *T. gondii* strains from South America and Asia were analysed using PCR-RFLP targeting ROP18, ROP5, ROP16 and ROP17 and the authors concluded that allele 1 of ROP18 in combination with allele 3 of ROP5 were key determinants of virulence in mice [6]. In the present study, ToxoDB genotype #13 (Groups 5 and 6) and ToxoDB genotype #6 (Group 8) were both associated with 100% mortality in mice; therefore, it was expected that they would have the virulent ROP profile. However, only ToxoDB genotype #6 had allele 1 of ROP18 and allele 3 of ROP5, whereas ToxoDB genotype #13 had allele 3 of ROP18 and allele 1 of ROP5. It may be the case that other genetic factors are involved in virulence or that although the virulent genotype profile applies to most virulent strains, it does not apply to all. For example, amongst the strains analysed by Shwab et al. [6], one was associated with 75% mortality in mice and had the same ROP18 and ROP5 alleles as ToxoDB genotype #13 in this study. Similarly, 39 out of 106 strains, accounting for 37% of those classified as lethal for mice, had allele 4 of ROP18 and allele 3 of ROP5. Also, out of 134 non-virulent isolates, only 9 had the ROP18/5 allelic combination of 3/1. Whole genome sequencing of virulent and avirulent genotype #13 isolates may help identify differences between the isolates and potentially other markers for virulence.

The isolates of intermediate virulence in this study had the same ROP profile except for one isolate which differed at the ROP17 locus. It is of note that this isolate (ToxoDB genotype #265), which had the same ROP17 allele as the avirulent isolates (ToxoDB genotypes #1 and #3), was associated with 30% mortality which is the threshold for designating an isolate as being of intermediate virulence (≥ 30% mortality) or avirulent (< 30% mortality). Perhaps there is a role for ROP17 in determining virulence, although this has not been described before. The ROP18/ROP5 allelic profile of 3/3 displayed by the isolates of intermediate virulence in this study is in accordance with previous studies where this allelic profile has been described for isolates causing varying levels of mortality [6]. The avirulent isolates had the same ROP profile with allele 2 at all loci, which is expected in Type II/Type II variant strains and has been described before [6].

Whilst it appears that ROP proteins play a role in determining virulence, it is likely to also involve a number of other parasite and host genetic factors. The majority of investigative studies into the mechanisms behind the conferrence of virulence by ROP proteins have been focused on mice but recent studies have shown that neither ROP18 nor ROP5 affected the survival of *T. gondii* in human cells [50]. In mice, ROP18 has been shown to phosphorylate and inactivate IFN-γ-induced IRGs thus preventing disruption of the parasitophorous vacuole and killing of the parasite; however, humans only have two IRG genes so it is unlikely that the same mechanism confers virulence in the human host [50].

## Conclusions

The results of this study have demonstrated that the ToxoDB genotype #13 isolates from the Caribbean displayed the same level of virulence as the ToxoDB genotype #6 from Brazil, resulting in 100% mortality of mice. The ToxoDB genotype #1 isolate from the Caribbean was similar to the avirulent European isolate (ToxoDB genotype #3). Whilst the isolates used in this study originated from animals, they represent the strains circulating in the environments of the Caribbean, Europe and South America which may cause disease in humans. Given the more severe sequelae associated with atypical strains it is imperative to understand their level of virulence so that disease outcome may potentially be predicted. This becomes even more important with the knowledge that it is possible for previously infected humans (and animals) to become re-infected with an atypical strain [17, 51]. A greater understanding of parasite virulence not only allows for the potential to predict disease outcome, it may also aid vaccine design, a more specific treatment regime as well as the development of new drug compounds [52]. Saraf et al. [10] recently called for a standardized methodology for determining *T. gondii* virulence in mice to allow for direct comparison between studies. Whilst this is much needed, it must also be recognised that ethical guidelines for working with laboratory animals differ between regions (particularly Europe and the Americas) and have to be taken into consideration when conducting experiments.
